# Design of a Direction-of-Arrival Estimation Method Used for an Automatic Bearing Tracking System

**DOI:** 10.3390/s16071145

**Published:** 2016-07-22

**Authors:** Feng Guo, Huawei Liu, Jingchang Huang, Xin Zhang, Xingshui Zu, Baoqing Li, Xiaobing Yuan

**Affiliations:** 1 Science and Technology on Microsystem Laboratory, Shanghai Institute of Microsystem and Information Technology, Chinese Academy of Sciences, Shanghai 200050, China; liuhuawei@mail.sim.ac.cn (H.L.); zuxs@mail.sim.ac.cn (X.Z.); libq@mail.sim.ac.cn (B.L.); yuanxb@mail.sim.ac.cn (X.Y.); 2 IBM-Research China Lab, Shanghai 201203, China; jchhuang@mail.ustc.edu.cn (J.H.); zxinscholar@gmail.com (X.Z.)

**Keywords:** DOA estimation, bearing tracking, narrowband, wideband, sub-band magnitude-squared coherence, microphone array

## Abstract

In this paper, we introduce a sub-band direction-of-arrival (DOA) estimation method suitable for employment within an automatic bearing tracking system. Inspired by the magnitude-squared coherence (MSC), we extend the MSC to the sub-band and propose the sub-band magnitude-squared coherence (SMSC) to measure the coherence between the frequency sub-bands of wideband signals. Then, we design a sub-band DOA estimation method which chooses a sub-band from the wideband signals by SMSC for the bearing tracking system. The simulations demonstrate that the sub-band method has a good tradeoff between the wideband methods and narrowband methods in terms of the estimation accuracy, spatial resolution, and computational cost. The proposed method was also tested in the field environment with the bearing tracking system, which also showed a good performance.

## 1. Introduction

The automatic tracking of vehicles is an important task for the wireless sensor network (WSN), especially for the unattended ground sensors (UGS). Moreover, the bearing tracking is also the basic requirement for a single sensor [1]. However, many factors increase the difficulty of designing an automatic bearing tracking system. These factors include the limited processing and power resources, small aperture, and the harsh work environment [2,3,4,5].

To make the sensors portable and durable, we designed the system with the principles of small size and low complex hardware structure. With the help of the MEMS microphones, we have finished the design of the hardware system in the previous works [6,7]. Moreover, the automatic detection of vehicles has also been addressed in [8]. However, both the small aperture and the limited processing resources hinder the implementation of the bearing tracking algorithms. To get a satisfying bearing tracking performance, a good direction-of-arrival (DOA) estimation method is crucial. In this paper, we mainly focus on the design of the DOA estimation method suited to this automatic bearing tracking system.

The DOA estimation methods can be divided into two categories: the narrowband methods and the wideband methods [9]. With a high DOA estimation accuracy and low computational cost, the narrowband methods are widely used in the wireless sensors. Among them, the multiple signal classification (MUSIC) is very popular for its good performance [10]. However, despite a high spatial resolution for the narrowband signals, the resolution will suffer much loss for the signals of vehicles which are not narrowband [11,12]. Furthermore, the small aperture of the microphone array used on the UGS would also exacerbate this problem [13,14,15].

For the UGS to track multiple vehicles, a high resolution is important. To get a high resolution, the wideband DOA estimation methods are promising [16]. The wideband methods could be divided into the incoherent and coherent methods. The incoherent methods divide the frequency band into multiple sub-bands, then estimate the DOA on each sub-band by narrowband methods, and combine the results at last [17]. However, it is a heavy computational burden to conduct the DOA estimation on every sub-band and the efficiency of these methods deteriorates for both closely distributed sources and low SNR [18]. Unlike the incoherent methods, the coherent methods use a focusing transformation matrix to focus the sub-bands on the chosen sub-band and then estimate the DOA on the focused sub-band by narrowband methods [16]. Among the coherent wideband methods, the two-sided correlation transformation (TCT) is common [19]. However, since the TCT needs many eigenvalue decomposition (EVD) operations whose computational complexity is $O(M^{3})$ for an M×M matrix [12], its computational cost may also be high for wireless sensors with long time monitoring. Besides, the coherent methods may be less accurate than the incoherent methods for ground vehicles in the wild [12,16]. The field experiments also show that the TCT is vulnerable in the wild environment.

Since the broad frequency band of the signals would reduce the spatial resolution of the narrowband DOA estimation methods, inspired by wideband methods, we want to choose a sub-band from the wideband signals and use it for the DOA estimation, expecting to achieve a good resolution. In the wild environment, the factors including the wind noise, signal attenuation, and meteorological variations could reduce the performance of the DOA estimation methods. The wind noise is one of the major factors [20]. Many researches show that the wind noise is nearly incoherent [21,22]. Thus, Zhang et al. [6] used the spatial coherence to select the frequencies less affected by the wind noise and yielded a good result. In the method, the spatial coherence of each frequency bin was measured by the magnitude-squared coherence (MSC) [23,24].

However, Zhang et al. [6] needs a threshold to select the available frequencies and an appropriate threshold is very difficult to choose in the real environment. Since this method would lead to the frequency band with a variable width, it brings more difficulty for the target detection [8]. Furthermore, this method is still a narrowband method, as the chosen frequencies are usually too wide to be narrowband, this method would also suffer a low resolution. In the previous work, as we just took the DOA estimation of a single vehicle into consideration, the resolution was paid less attention and the narrowband method could also be a choice for its low computational cost. Since we employ the system for bearing tracking, a good resolution is preferred. Therefore, a sub-band approach is promising to the bearing track system. Although the MSC is famous, its extension to sub-band has not been studied and the previous works almost concentrated on its estimation [25,26]. Thus, we extend the MSC to the sub-band and propose the sub-band magnitude-squared coherence (SMSC) to measure the coherence between the frequency sub-bands of wideband signals. The SMSC could reflect the degrees how much the sub-band is affected by the noise. The simulation shows that it is an effective measurement of the coherence between sub-bands. After the extension, we design a sub-band DOA estimation method for the vehicle bearing tracking of the wireless sensors with the help of SMSC.

It is necessary to note that a sub-band DOA estimation method was also mentioned in [27]. Xue et al. [27] and Wang et al. [28] connected the spectral estimation with the DOA estimation and then applied the spectral estimation methods to the DOA estimation. Among the spectral estimation methods, a sub-band method was proposed in [29]. However, this sub-band method used the wavelet packets to get the sub-band and then implemented the traditional spectral estimation method on each sub-band. Thus, the sub-band method in [27] is similar to the incoherent wideband methods and would bring a heavy computational burden. Furthermore, target detection is necessary for an automatic tracking system deployed in the wild environment. It is also a tough problem due to the limited resources [30]. We suggested a two-stage detection method for wireless sensors in the previous work [8]. In the work, the eigenvalues of the covariance matrix (CM) of the signals were used to conduct the second stage detection. As they were also the intermediate results of the subspace based DOA estimation algorithm, the second stage detection would improve the detection performance as well as not bring extra computational cost. As the final CM of signals will not be attained in both the incoherent wideband methods and the sub-band method proposed in [27], these methods are also not desirable for the this detection scheme.

Both simulations and field experiments were conducted to validate the designed sub-band method. The results show that in comparison with the famous wideband method TCT, this sub-band method is more robust to the uncorrelated noise as well as has a lower compuatational cost. While compared with the famous narrowband method MUSIC, the proposed method can achieve a much higher spatial resolution with only a little loss on the estimation accuracy. In other words, the proposed sub-band method can have a good tradeoff between the wideband methods and the narrowband methods. It is very much suited to the automatic bearing tracking system deployed in the field environment where a large amount of uncorrelated noise exists.

In general, this paper has the following contributions:

1. Introduce the design of an automatic bearing tracking system with a circular MEMS microphone array.
2. Extend the MSC to the sub-band and propose the SMSC to measure the coherence between the frequency sub-bands of wideband signals.
3. Design a sub-band DOA estimation method suitable for the bearing tracking system.

This paper is organized into five sections including the present one. Section 2 proposes the SMSC and briefly introduces the wideband DOA estimation methods which would be used in the third section. Section 3 introduces the design of the automatic bearing tracking system and proposes the sub-band DOA estimation method. In Section 4, the simulations are conducted to validate the proposed method. The field experiments are also implemented to test the designed bearing tracking system. Finally, Section 5 concludes this paper.

## 2. Sub-Band Magnitude-Squared Coherence

### 2.1. Definition of the SMSC

We first introduce the notations used in this paper.

1. The superscript * denotes the conjugate of the complex number.
2. The text in bold denotes vectors.
3. The · denotes the matrix multiplication.
4. The superscript *H* denotes the conjugate transpose of the matrix.
5. The italic E denotes the expectation.

Assume X(f) and Y(f) are the discrete fourier transform (DFT) of the signals X(n) and Y(n) respectively. We divide the frequency band into *J* identical sub-bands and remove the mean of each sub-band in the frequency domain. Then we use random variables $x(f_{i})$ and $y(f_{i})$, i∈[1,J] to represents the sub-bands, in which $f_{i}$ represents the center frequency (CF) of the *i*th sub-band. The frequency bins of the *i*th sub-band are regarded as the sampling values of the variables. Thus, the *i*th subband’s sub-band magnitude-squared coherence (SMSC) between X(n) and Y(n) is defined as:

(1) $$r_{xy}\left(f_{i}\right)=\frac{p_{xy}{\left(f_{i}\right)}^{*}p_{xy}\left(f_{i}\right)}{p_{xx}\left(f_{i}\right)p_{yy}\left(f_{i}\right)}$$

in which $p_{xy}\left(f_{i}\right)=E\left\{x{\left(f_{i}\right)}^{*}y\left(f_{i}\right)\right\}$ represents the cross power between $x(f_{i})$ and $y(f_{i})$ while $p_{xx}\left(f_{i}\right)$ and $p_{yy}\left(f_{i}\right)$ represent the powers of $x(f_{i})$ and $y(f_{i})$ respectively. Specially, if *J* is equal to the number of frequency bins and the operation of removing the mean is abandoned, the SMSC degrades to the MSC.

If $x\left(f_{i}\right)=e^{-j\omega_{0}\tau }y\left(f_{i}\right)$ in which $\omega_{0}$ and *τ* are two constants and $j=\sqrt{-1}$, $p_{xy}\left(f_{i}\right)=E\left\{x{\left(f_{i}\right)}^{*}y\left(f_{i}\right)\right\}=e^{j\omega_{0}\tau }p_{yy}\left(f_{i}\right)$. Thus

(2) $$r_{xy}\left(f_{i}\right)=\frac{e^{-j\omega_{0}\tau }p_{yy}{\left(f_{i}\right)}^{*}e^{j\omega_{0}\tau }p_{yy}\left(f_{i}\right)}{p_{yy}\left(f_{i}\right)p_{yy}\left(f_{i}\right)}=1$$

While since the mean of each sub-band is removed, $E\left\{x\left(f_{i}\right)\right\}=E\left\{y\left(f_{i}\right)\right\}=0$. If $x(f_{i})$ and $y(f_{i})$ are uncorrelated,

(3) $$p_{xy}\left(f_{i}\right)=E\left\{x{\left(f_{i}\right)}^{*}y\left(f_{i}\right)\right\}=E\left\{x{\left(f_{i}\right)}^{*}\right\}E\left\{y\left(f_{i}\right)\right\}=0$$

Hence,

(4) $$\begin{array}{c} r_{xy}\left(f_{i}\right)=1,\;\;if\;\;x\left(f_{i}\right)=e^{-j\omega_{0}\tau }y\left(f_{i}\right)\\ r_{xy}\left(f_{i}\right)=0,\;\;if\;\;x\left(f_{i}\right)\;and\;y\left(f_{i}\right)\;are\;uncorrelated \end{array}$$

### 2.2. The Estimation of the SMSC

Similar to the estimation of the MSC [23], the steps to estimate the $r_{xy}\left(f_{i}\right)$ are:

1. Divide the signals X(n) and Y(n) into *L* identical sized blocks, respectively.
2. Each block is processed by FFT to get the frequency bins. Divide the frequency bins into *J* identical sub-bands and *K* is the number of frequency bins of each sub-band. If ${\bar{X}}_{l}^{i}\left(K\right)$ represents the frequency bins of ith sub-band of the lth block after removing the mean and it is a *K*-dimension column vector, then we have
  (5) $$\begin{array}{ccc} {\hat{p}}_{xy}{\left(f_{i}\right)}_{l} & = & \frac{1}{K}\left[{\bar{X}}_{l}^{i}{\left(K\right)}^{H}\cdot {\bar{Y}}_{l}^{i}\left(K\right)\right] \end{array}$$
  (6) $$\begin{array}{ccc} {\hat{p}}_{xx}{\left(f_{i}\right)}_{l} & = & \frac{1}{K}\left[{\bar{X}}_{l}^{i}{\left(K\right)}^{H}\cdot {\bar{X}}_{l}^{i}\left(K\right)\right] \end{array}$$
  (7) $$\begin{array}{ccc} {\hat{p}}_{yy}{\left(f_{i}\right)}_{l} & = & \frac{1}{K}\left[{\bar{Y}}_{l}^{i}{\left(K\right)}^{H}\cdot {\bar{Y}}_{l}^{i}\left(K\right)\right] \end{array}$$
3. Finally, the SMSC of the *i*th sub-band $r_{xy}\left(f_{i}\right)$ is estimated by Equation (8).
  (8) $$r_{xy}\left(f_{i}\right)=\frac{1}{L}\sum_{l=1}^{L}\frac{{\hat{p}}_{xy}{\left(f_{i}\right)}_{l}^{*}{\hat{p}}_{xy}{\left(f_{i}\right)}_{l}}{{\hat{p}}_{xx}{\left(f_{i}\right)}_{l}{\hat{p}}_{yy}{\left(f_{i}\right)}_{l}}$$

**Figure 1:**
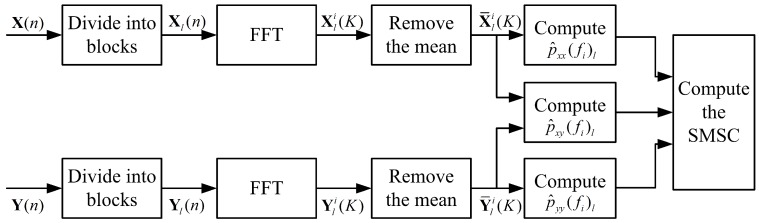
The flow chart to estimate the sub-band magnitude-squared coherence (SMSC).

The flow chart to estimate the $r_{xy}\left(f_{i}\right)$ is shown in Figure 1.

### 2.3. The Wideband Methods

Consider an *M*-element array receives *P* wideband signals. Its mathematical model in the frequency domain is

(9) $$X\left(f_{i}\right)=A\left(f_{i}\right)\cdot S\left(f_{i}\right)+N\left(f_{i}\right)$$

in which $X(f_{i})$, $S(f_{i})$, and $N(f_{i})$ represent the *i*th frequency sub-bands of the received signals, the original signals, and the noise respectively [31]. $A\left(f_{i}\right)=\left[a_{1}\left(f_{i}\right),a_{2}\left(f_{i}\right),\cdots ,a_{P}\left(f_{i}\right)\right]$ is the manifold matrix and $a_{p}\left(f_{i}\right)={\left[exp\left(-j2\pi f_{i}\tau_{1p}\right),\cdots ,exp\left(-j2\pi f_{i}\tau_{mp}\right),\cdots ,exp\left(-j2\pi f_{i}\tau_{Mp}\right)\right]}^{T}$, p∈[1,P] is the steering vector, where $\tau_{mp}$ is the time delay of the *p*th signal between the *m*th microphone and the reference microphone. Then the steps of the coherent wideband methods are [16,19]:

1. Divide the received signals X(n) into *L* identical sized blocks to get $X_{l}\left(n\right),l\in \left[1,L\right]$. And then the frequency sub-band $X_{l}^{i}\left(K\right)$ is acquired by dividing the frequency domain into *J* identical sub-bands after the FFT of $X_{l}\left(n\right)$. It is necessary to note that $X_{l}^{i}\left(K\right)$ is an M×K matrix. Estimate the CM $R_{XX}\left(f_{i}\right)$ of each sub-band by Equation (10).
  (10) $${\hat{R}}_{XX}\left(f_{i}\right)=\frac{1}{L}\sum_{l=1}^{L}X_{l}^{i}\left(k\right)\cdot X_{l}^{i}{\left(k\right)}^{H},i\in \left[1,J\right]$$
2. Select the focusing frequency (FF) $f_{0}$ and compute the focusing transformation matrix $T(f_{i})$ for each sub-band, where the $f_{0}$ can be the central frequency of the chosen focusing sub-band and $T(f_{i})$ is the solution of $T\left(f_{i}\right)A\left(f_{i}\right)=A\left(f_{0}\right)$.
3. Construct the CM $R_{0}$ at the FF through the focusing transformation.
  (11) $$R_{0}=\frac{1}{J}\sum_{i=1}^{J}T\left(f_{i}\right)R_{XX}\left(f_{j}\right)T^{H}\left(f_{i}\right)$$
4. Apply MUSIC [10] or other DOA estimation methods to estimate the DOA by $R_{0}$.

In comparison with the narrowband methods, these wideband methods could achieve a high spatial resolution by dividing the broad frequency band into the sub-bands. However, the focusing transformation would cost much processing resource [12,31].

## 3. Design of the Bearing Tracking System

### 3.1. Hardware Architecture of the Bearing Tracking System

**Figure 2:**
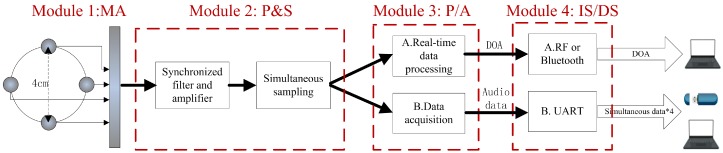
System architecture of the automatic bearing tracking system.

The automatic bearing tracking system should proceed the bearing tracking in real-time and then send out the bearing information. Moreover, as a prototype, the system can collect the audio data for the design of the bearing tracking algorithm. The block diagram of the automatic bearing tracking system is shown in Figure 2.

The system can be divided into four modules by function: microphone array (MA), preprocessing and sampling (P&S), real-time data processing or data acquisition (P/A), and information-sending or data storage (IS/DS). The acoustic signals from the MA module are sampled into four simultaneous digital signals by module P&S. Then the system could be used as either the real-time data processing to the bearing tracking or the data acquisition to the design of the bearing tracking algorithm in module P/A. Finally, the bearing tracking sensor either sends out the DOA of the monitored vehicle to the receiver by the bluetooth or the radio frequency (RF) or just stores the collected audio data in the memory device in the IS/DS module.

**Figure 3:**
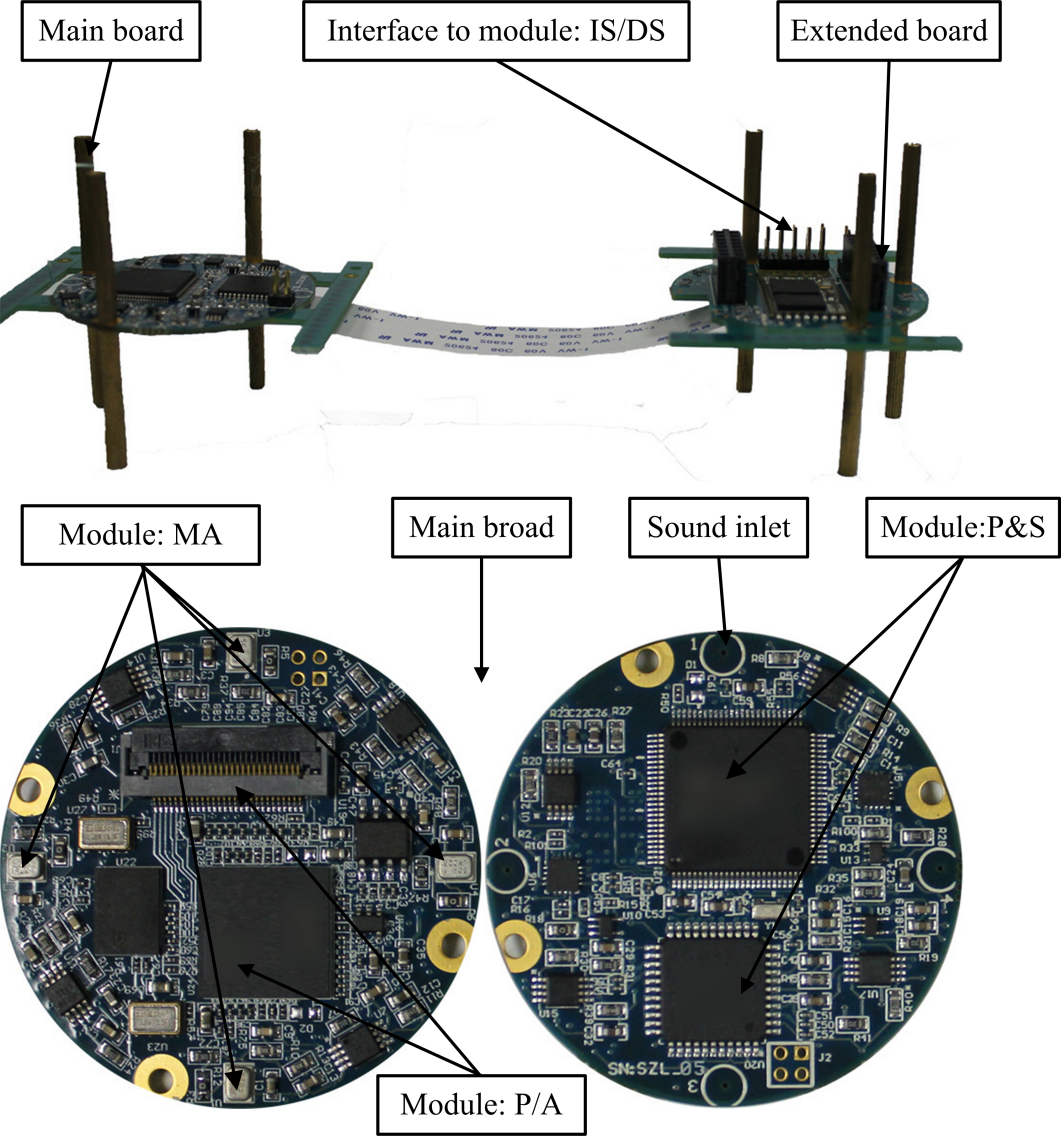
Photograph of the automatic bearing tracking system.

To make sure that the array has the same resolution in all directions, we use the circular array. Furthermore, the maximum distance between two adjacent microphones should be less than half the wavelength to avoid the space aliasing. Considering vehicle signals with the frequency band from 50 Hz to 3000 Hz [20,32], the maximum distance should be less than 5.6 cm. By using the MEMS microphone array, the bearing tracking system can achieve such a small size. The diameter of the MEMS microphone array we used is 4 cm, which makes the system portable and easy to cover. The audio signal is sampled at the rate of 8192 Hz. The photograph of the bearing tracking system is shown in Figure 3.

Four MEMS microphones (ADMP504) are employed to capture the acoustic signals. Then, a 4-channel 16-bit simultaneous ADC and supplemental hardware circuit are used to get the digital signals. The DSP (ADSP21375) is chosen for the real-time data processing. When deployed in the field environment, the sensor would run the two-stage detection algorithm proposed in [8] until a vehicle invasion is confirmed. After the automatic detection of the invasion, the sensor will estimate the DOA of the invading vehicle and send out the tracking information in real-time by IS/DS module.

### 3.2. The Sub-Band DOA Estimation

Although the small aperture makes the sensor portable and hard to discover, it would exacerbate the spatial resolution loss of the narrowband methods [13,15]. Inspired by the wideband methods, we try to choose a sub-band from the wideband signal for the DOA estimation to get a high spatial resolution and discard the focusing transformation to save more computational resource.

Denote the *i*th sub-band of the signal received by the *n*th microphone and the *i*th sub-band of the same signal received by the *m*th microphone as $X_{n}\left(f_{i}\right)$ and $X_{m}\left(f_{i}\right)$, respectively. Both the sub-bands are assumed to be narrowbands. Then with noise absent,

(12) $$X_{n}\left(f_{i}\right)=e^{-j2\pi f_{i}\left(\tau^{nm}\right)}X_{m}\left(f_{i}\right)$$

in which $\tau^{nm}$ is the time delay of the signal between the *m*th microphone and the *n*th microphone. If ${\bar{X}}_{n}\left(f_{i}\right)$ and ${\bar{X}}_{m}\left(f_{i}\right)$ are got by removing the means of $X_{n}\left(f_{i}\right)$ and $X_{m}\left(f_{i}\right)$ respectively,

(13) $${\bar{X}}_{n}\left(f_{i}\right)=e^{-j2\pi f_{i}\left(\tau^{nm}\right)}{\bar{X}}_{m}\left(f_{i}\right)$$

Use random variables $x(f_{i})$ and $y(f_{i})$ to represent ${\bar{X}}_{n}\left(f_{i}\right)$ and ${\bar{X}}_{m}\left(f_{i}\right)$, respectively and regard the frequency bins of the sub-band as the sampling values of the variables. Thus, $x\left(f_{i}\right)=e^{-j2\pi f_{i}\left(\tau^{nm}\right)}y\left(f_{i}\right)$. According to Equation (4), the SMSC of the sub-band is 1. Furthermore, there is a narrowband condition shown in Equation (14) [33].

(14) $$sinc(B\tau^{1m})≅1$$

where *B* is the bandwidth. Even if the signal received by our microphone array has a frequency band of [0,4096] Hz, with dividing the signal into only 4 sub-bands, each sub-band could be regard as narrowband with the value of 0.98. Thus, the SMSCs between the two signals from any two microphones would be close to 1 with the noise absent after dividing the signal into no less than 4 sub-bands. Therefore, we can measure the degrees to which the sub-band is affected by the noise through the SMSC. Afterwards, we could select the appropriate sub-band by SMSC. After the selection of the sub-band, a narrowband method can then be used for the DOA estimation. The steps of the designed sub-band DOA estmation method are:

1. Select the signals from any two microphones and compute the SMSCs of each frequency sub-band of the signals. Then, choose the sub-band with the largest SMSC as the DOA estimation sub-band.
2. Estimate the CM ${\hat{R}}_{XX}\left(f_{i}\right)$ of the chosen sub-band by Equation (10).
3. Attain the number of acoustic emitters by ${\hat{R}}_{XX}\left(f_{i}\right)$, according to some signal number estimation criterion such as the MDL [34].
4. Apply MUSIC or other DOA estimation methods to estimate the DOA by ${\hat{R}}_{XX}\left(f_{i}\right)$.

**Figure 4:**
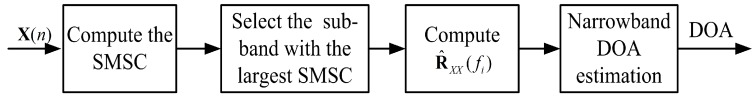
The flow chart of the sub-band direction-of-arrival (DOA) estimation method.

The flow chart of the proposed sub-band method is shown in Figure 4. In particular, although the proposed method is used for our bearing tracking system with a circular array, it can also be used to other array geometries with their corresponding steering vectors. Since we must ensure that the narrowband condition is satisfied with dividing the sub-bands, the $\tau^{1m}$ should be small. As the UCA (uniform circular arary) has the minimum size in the planar arrays when the interelement spacing is fixed, this sub-band method is best suited to the UCA. For instance, if the interelement spacing is $\sqrt{2}$ cm, to the array with 4 elements, the maximum distance between the elements is 2 cm for the UCA and $3\sqrt{2}$ cm for the ULA (uniform linear arary). The computational costs of the MUSIC, TCT, and the sub-band method are all $O(M^{3})$ but the TCT has a multiply value of *J* that is $O(J*M^{3})$ which causes it to be more sensitive to changes in *M*. In comparison with the wideband methods, the sub-band DOA estimation method abandons the focusing transformation and only makes use of one sub-band of the signals to save the computational resource as much as possible.

## 4. Simulations and Experiments

### 4.1. Simulations

**Figure 5:**
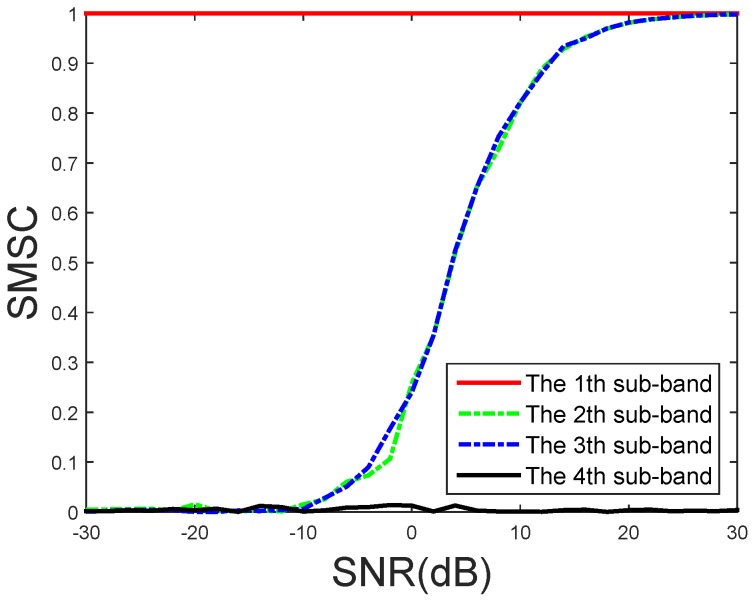
The SMSC under different SNRs.

A simulation is conducted to validate Equation (4). A linear frequency modulation signal (LFMS) with 3 KHz bandwidth is received by our microphone array. The wideband signal is divided into four sub-bands. The first sub-band is not contaminated by any noise at all while the fourth sub-band has the noise only. The uncorrelated Gaussian noise with different SNRs is appended on the second and third sub-bands. The SMSC is computed by the signals from two randomly chosen microphones and L=1. The results in Figure 5 validate that the SMSC is 1 when only signal is received by the microphone array while it would be zero if only uncorrelated noise exists. Figure 5 also indicates that the SMSC can be used to select the sub-band with the highest SNR.

**Figure 6:**
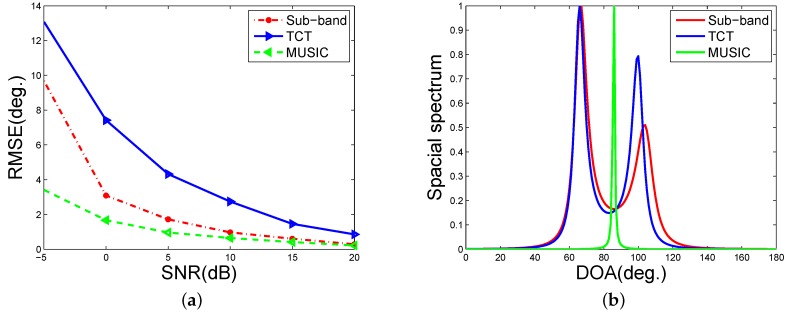
Performance comparison. (**a**) The RMSEs of the three DOA estimation methods; (**b**) Spatial spectrums of the three DOA estimation methods.

Consider a LFMS with bandwidth [50,3000] Hz is received by the microphone array with a diameter of 4 cm. The uncorrelated noise are added to test the robustness. The SNR is computed by $10log10(P_{s}/P_{n})$, where $P_{s}$ and $P_{n}$ are the signal energy and noise energy in the entire band, respectively. As the sub-band method is a tradeoff between the wideband methods and the narrowband methods. To make a better demonstration of the sub-band method, we give comparisons with both the narrowband methods and the wideband methods. We compare the sub-band method with the well-known wideband method TCT and the popular narrowband method MUSIC. Besides, the MUSIC is applied to the final DOA estimation step of both the TCT and the sub-band method. We evaluate the performance by RMSE (root-mean-square error). The RMSE is computed as $RMSE=\sqrt{E(|\theta -\hat{\theta }{|}^{2})}$ in 1000 Monte Carlo experiments, where $\hat{\theta }$ is the estimation of the DOA *θ*. Moreover, without any further statement, L=2, J=4, the time length is 0.125 s, and the sampling rate is 8192 Hz in the following experiments including the current one. The results are shown in Figure 6a. Figure 6a indicates that the MUSIC outperforms the TCT method in the uncorrelated noise condition while the sub-band method is consistently between them in terms of the estimation accuracy. When there are two LFMSes with bandwidth [50,3000] Hz and [70,3100] Hz from 70 degrees to 100 degrees respectively and an uncorrelated noise with a SNR of 15 dB is added, the spatial spectrums are shown in Figure 6b. This demonstrates that the TCT yields the highest resolution while the narrowband MUSIC suffers a bad performance and the sub-band method is almost comparable to the TCT.

**Figure 7:**
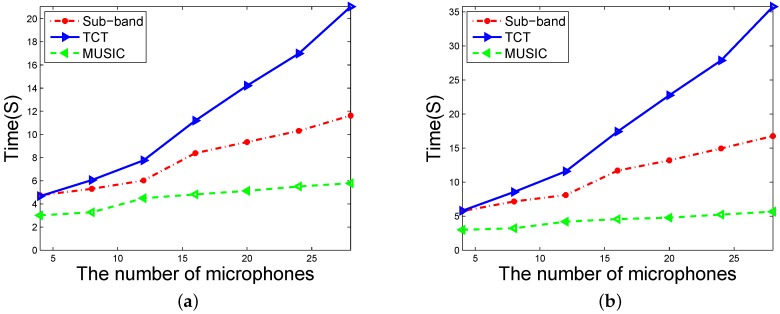
The time elapse of the three DOA estimation methods under 1000 estimations. (**a**) J=8; (**b**) J=16.

To further improve the spatial resolution and reduce the estimation error, the most convenient method is increasing the number of microphones. The computational complexity of the increasing is compared in Figure 7. The LFMS with the bandwidth of 3 KHz is received by the microphone arrays with different numbers of microphones. All the evaluations were performed in the Matlab 2014b on a computer (dual core, 3.4 GHz CPU, and 8 GB memory). The results shown in Figure 7 indicate that the MUSIC method is the most computationally efficient and grows the slowest as the number of microphones employed increases. The TCT method has the worst computational time efficiency which grows at an increasing rate as the number of microphones increases. The proposed sub-band method’s computational complexity is consistently between these two methods. The simulation results agree with the previous computational cost analysis. The results indicate that the sub-band method is more convenient for the bearing tracking system to increase the number of the microphones than the TCT method.

### 4.2. Field Experiments

**Figure 8:**
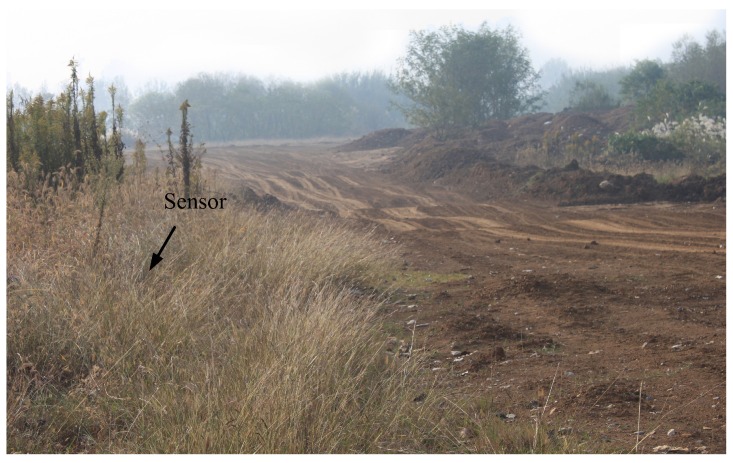
The environment of the field experiment.

Audio signals of vehicles were gathered to validate the performance of the bearing tracking system. The environment of the field experiment is shown in Figure 8. Vehicles were passing away the sensor. The DOA of the passing vehicle satisfies $\theta =\arctan(\left(v\left(t-t_{0}\right)\right)/d)$, where *θ* is the DOA of the vehicle, *v* is the speed of the vehicle, and *d* is the distance from the microphone array to the closest point of approach (CPA) [6]. We compared the sub-band method with both the narrowband MUSIC and the TCT. Moreover, the proposed method in the previous work [6] achieved a good performance for the DOA estimation of a single vehicle in the field experiment with the help of MSC (We call it MSC-MUSIC for brevity.). Although this method is still a narrowband method, we also made a comparison with it.

**Figure 9:**
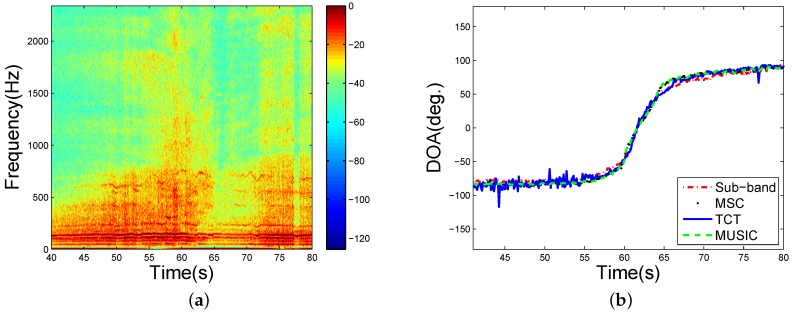
Single vehicle tracking. (**a**) is the spectrum; (**b**) is the estimated DOAs.

At first, we evaluated the performance of tracking a single vehicle. In the single vehicle tracking case, a tracked vehicle was passing over the sensor and the wind speed was less than 2 m/s. Its spectrum is shown in Figure 9a. It shows that the signal of the tracked vehicle has a broad frequency band and its energy distributes unevenly on the band. We performed the MUSIC, MSC-MUSIC, TCT, and the designed sub-band method on the tracked vehicle signal. The signals were divided into frames, the frame length was 1024, and there was no overlap between frames. The bandwidth used in the sub-band method and TCT was [50,3000] Hz. The results are shown in Figure 9b. The four DOA estimation methods had the similar performance when the vehicle was close to the sensor. The performance of TCT would suffer some loss when the vehicle was far from the sensor while the sub-band method and the narrowband methods could still achieve a steady bearing tracking. The results indicate that the sub-band method and the MUSICs have a better DOA estimation performance than the TCT for the single vehicle tracking. Besides, the bearing tracking curves of the MSC-MUSIC and the MUSIC are nearly the same in this case.

**Figure 10:**
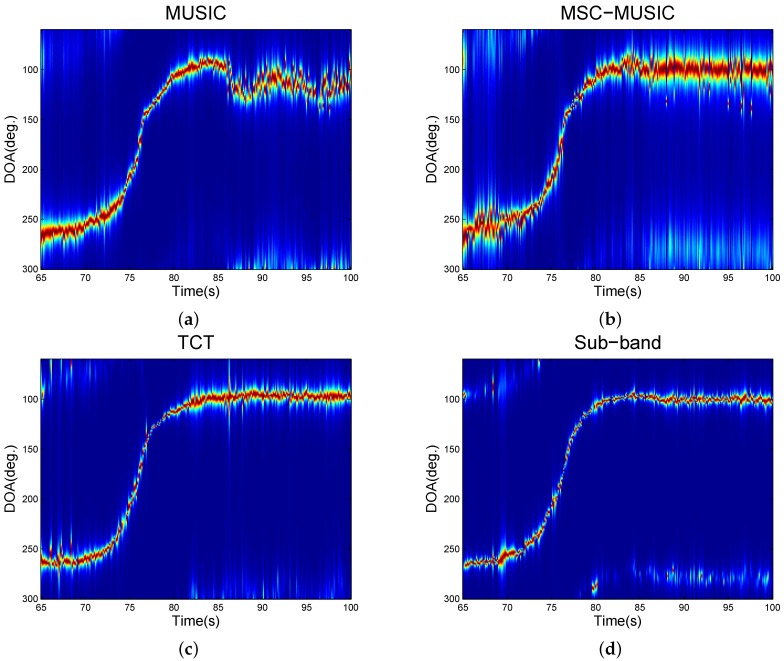
Vehicle tracking under interferences. (**a**) is from the multiple signal classification (MUSIC); (**b**) is from the magnitude-squared coherence (MSC)-MUSIC; (**c**) is from the two-sided correlation transformation (TCT); (**d**) is from the proposed sub-band method.

Then, we evaluated the tracking ability under acoustic emitter interferences. A tracked vehicle remained in a place with its engine on to act as an interference; then another tracked vehicle were moving towards the interference. The wind speed was less than 2 m/s . The bearing tracking results are shown in Figure 10. Figure 10 indicates that the sub-band method and the TCT could give a good tracking for the whole moving trajectory while the MUSIC had a big fluctuation when the vehicle approached the interference emitter. Besides, although the MSC-MUSIC could also give a steady tracking with the MSC to choose available frequency bins, this narrowband method would suffer a bad resolution.

**Figure 11:**
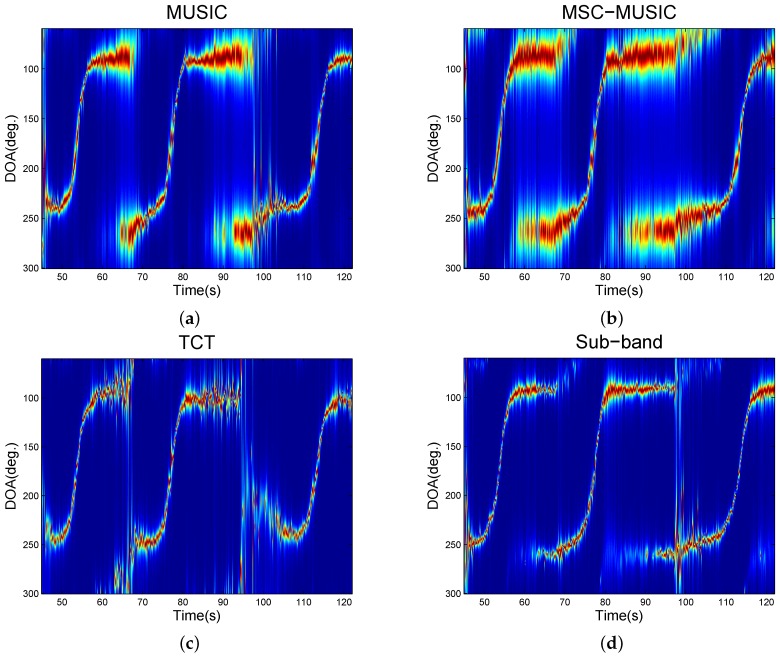
Multiple vehicles tracking. (**a**) is from the MUSIC; (**b**) is from the MSC-MUSIC; (**c**) is from the TCT; (**d**) is from the proposed sub-band method.

Three tracked vehicles were passing to test the ability of tracking multiple targets. A bad wind condition happened in the time duration [90,100] s with the wind speed of about 8 m/s. Results in Figure 11 show that the TCT and the sub-band method had thinner bearing tracking curves which indicated a better resolution in comparison with the narrowband methods. Furthermore, the MSC-MUSIC had a longer tracking than the MUSIC and the bearing tracking distance of TCT was much shorter than that of the sub-band method. The two facts indicate that the coherence in the frequency domain helps robustness and the sub-band method could achieve the best tracking in terms of both the robustness and resolution.

## 5. Conclusions

In this paper, we propose a DOA estimation method deployed in an automatic bearing tracking system. We firstly extend the MSC to the sub-band and present the SMSC to measure the coherence between frequency sub-bands. Furthermore, we design a sub-band DOA estimation method for vehicles in the field environment based on SMSC. In comparison with the narrowband method, the sub-band method tries to narrow the signal frequency band to get a high resolution. Compared with the wideband method, the proposed method chooses the sub-band with the least noise influence and discards the focusing process to reduce the computational cost as much as possible. The simulations show that the sub-band method is a good tradeoff between the wideband methods and the narrowband methods in terms of the DOA estimation accuracy, computational cost, and spatial resolution. The field experiments were also conducted to test the proposed sub-band method. The results demonstrate that with the proposed sub-band method, the bearing tracking system could achieve a satisfying performance for vehicles in the field environment.
